# A thermophone-based FM sonar for airborne ultrasonic sensing under drone flight noise

**DOI:** 10.1038/s41598-026-65909-3

**Published:** 2026-09-10

**Authors:** Yasufumi Yamada, Kanta Hasegawa, Yuma Watabe, Shinichi Sasaki, Takaaki Asada, Shizuko Hiryu

**Affiliations:** 1 Department of Complex and Intelligent Systems, Future University Hakodate, Hakodate, Hokkaido 041-8655 Japan; 2 Program of Mathematical and Life Sciences, Hiroshima University, Higashi-Hiroshima, Hiroshima 739-8511 Japan; 3 Faculty of Life and Medical Sciences, Doshisha University, Kyotanabe, Kyoto 610-0394 Japan; 4 Murata Manufacturing Co., Ltd., Yasu, Shiga 520-2393 Japan; 5 Acoustic Navigation Research Center, Doshisha University, Kyotanabe, Kyoto 610-0394 Japan

**Keywords:** Thermophone, LPM signal, Ultrasonic sensing, Cheap robust design, UAV, Echolocation, Engineering, Physics

## Abstract

Bats achieve agile navigation, including obstacle avoidance and prey pursuit in complex environments, using a remarkably simple active sonar system consisting of a single sound emitter and two receivers. Their biosonar highlights the potential of broadband FM pulses in simple sensing architectures. Here, we present a bat-inspired FM sonar system for drones, employing a single ultrasonic transmitter and two receivers. The system integrates a lightweight thermoacoustic emitter (thermophone) capable of generating broadband frequency-modulated (FM) pulses, including echolocation-like signals. Owing to its resonance-free operation, the thermophone provides wideband ultrasonic emission with low voltage input. Using onboard cross-correlation processing, we demonstrate object detection, two-dimensional localization, and low-speed two-dimensional obstacle avoidance using ultrasound alone during autonomous drone flight. The proposed system successfully detects narrow obstacles even under flight conditions in which the raw onboard microphone signals are dominated by drone flight-induced noise. Our results demonstrate the feasibility of thermophone-based FM sonar for airborne ultrasonic sensing during drone flight and establish thermophone technology as a promising platform for future bat-inspired aerial sonar system.

**Supplementary Information:**

The online version contains supplementary material available at https://doi.org/10.1038/s41598-026-65909-3.

## Introduction

Autonomous navigation in unmanned aerial vehicles (UAVs) has advanced substantially, driven by improvements in sensor fusion and lightweight onboard computing^1–3^. Vision-based sensing, in particular, has become the dominant strategy for both indoor and outdoor navigation, enabling drones to detect obstacles, follow trajectories, recognize landmarks, and perform complex maneuvers^4–7^. At the same time, interest in simple ultrasonic sensing systems has continued to grow. In addition to ground vehicle platforms employing a minimal architecture consisting of one transmitter and two receivers^8,9^, similar approaches have increasingly been adopted for aerial robots^10–12^. More recently, simple ultrasonic drone systems have been reported to operate in challenging environments such as fog, snow, and forests^10^. A similarly minimal sensing configuration is found in biosonar system of bats. Many bats emit ultrasonic pulses from the mouth or nostrils and extract spatial information from returning echoes at both ears, a process termed echolocation^13,14^. With only a single sound emitter and two receivers, bats successfully perform diverse behaviors such as obstacle avoidance^15–17^, prey capture^18–21^, landing^22,23^, and coordinated group flights^24,25^. These engineering and biological capabilities suggest that simple active sonar designs have the potential to operate across a wide range of environments.

The majority of echolocating bat species employ FM calls. Their multi-harmonic FM pulses span tens to hundreds of kHz^26,27^ and reach source levels of 110–130 dB SPL^28^, helping maintain detectable echo levels despite atmospheric attenuation and providing wide beam coverage (± 20–50° half-amplitude;^29–33^). Building on this strong acoustic baseline, bats further adapt their spatial sensing strategy by dynamically adjusting pulse intensity, time–frequency structure^20,34,35^, and beam width^18,36^ to meet specific flight demands. Previous bio-inspired sonar studies also have demonstrated applications such as obstacle avoidance^8,9,37^, foliage detection^9^, and passageway mapping^38^, but most implementations have been restricted to ground vehicles or stationary platforms. Airborne systems that employ a broadband ultrasonic FM pulse remain exceedingly rare. A major limitation is transmitter performance: generating broadband, high-intensity pulses while maintaining robustness against drone flight-induced noise is difficult under strict payload and power constraints. Together, these characteristics highlight a fundamental gap between biological and artificial airborne sonar systems.

To address this gap, we focused on the thermoacoustic transducer (Thermophones) that emit sound via air vibration caused by periodic heating and cooling of a metal plate synchronized with the input power^39,40^. Because no mechanical resonance is involved, thermophones can generate broadband signals. In this study, we developed an autonomous aerial robot (drone) fully equipped with a lightweight onboard active sonar system, consisting of a thermophone-based broadband ultrasonic emitter^39,40^ and two MEMS microphones. Using this sonar-equipped drone platform, we demonstrate obstacle detection, two-dimensional localization, and low-speed two-dimensional obstacle avoidance during autonomous drone flight, thereby evaluating the feasibility of thermophone-based FM sonar for airborne ultrasonic sensing.

## Results

### Overview of the proposed active sonar system for drone

**Fig. 1:**
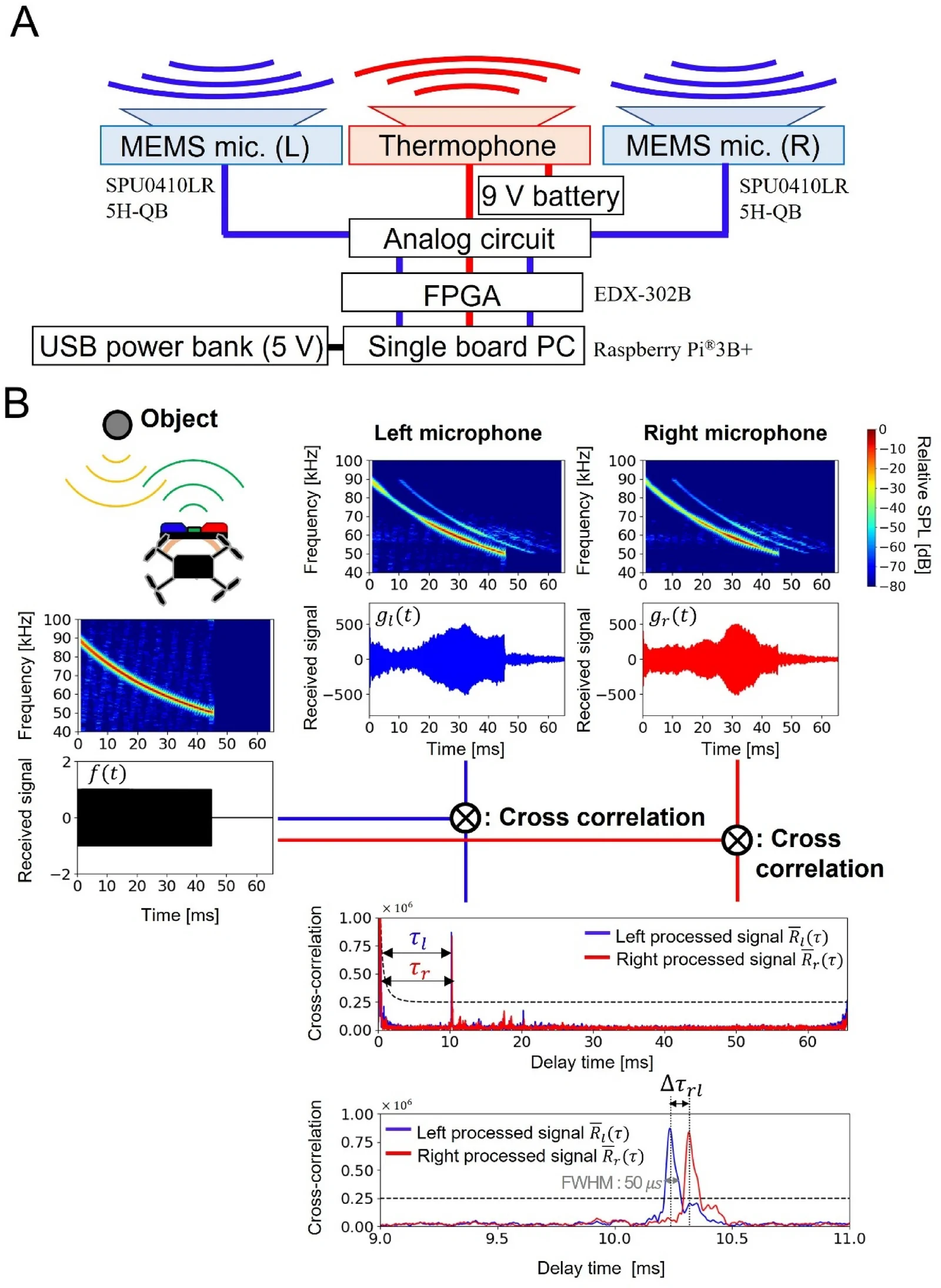
Onboard active sonar system using a thermophone and two microphones. (**A**) Schematic of the hardware configuration, including the thermophone, MEMS microphones, analog circuit, FPGA, and Raspberry Pi system. (**B**) Signal processing flow for acoustic sensing using cross-correlation. Example signals were recorded with a target placed in front of the left microphone under static conditions. Echo arrival times were extracted from the envelopes of the cross-correlation function (right and left channels) when they exceeded a predefined threshold (black dashed line). The cross-correlation echo envelope exhibited a FWHM of 50 µs, corresponding to a theoretical ranging resolution of approximately 17 mm. Target distance was calculated from the average echo delay, and horizontal direction was estimated from interaural time differences $\:\varDelta\:{\tau\:}_{rl}$.

Our constructed autonomous ultrasonic sensing system consists of a thermophone, two MEMS microphones (Knowles, SPU0410LR5H-QB), an analog circuit, a field programable gate array (XILINX, EDX-302B), a single board PC (Raspberry Pi, Model 3B+), a 9v dry cell battery, and a 5v USB power bank. An overview is shown in Fig. 1A. The total mass of the onboard system was 343 g. The two microphones were mounted 10 cm apart, with the thermophone positioned at the center. Each received signal was amplified (+ 46 dB) and bandpass filtered to flatten sensitivity within ± 3 dB across 50–90 kHz. The FPGA served as both DA and AD converter while obstacle localization was performed on Raspberry Pi powered by a 5 V USB power bank.

The sampling frequency was set to 1 MHz to enable accurate estimation of obstacle distance and azimuth angle. Cross-correlation processing was applied to transmitted and received signals (Fig. 1B). The Full Width at Half Maximum (FWHM) of the cross-correlation echo envelope was 50 µs, corresponding to a theoretical distance resolution of approximately 17 mm. If an echo exhibited a certain time delay $\:\tau\:$ while preserving the transmitted waveform, the cross-correlation function $\:R\left(\tau\:\right)$ showed a distinct peak at $\:\tau\:$ .the maximum search time delay $\:{\tau\:}_{max}$ was set to 64 ms. To facilitate peak detection, the envelope of $\:R\left(\tau\:\right)$ was extracted using a Hilbert transform $\:\stackrel{-}{R}\left(\tau\:\right)$. Thresholding was applied to suppress spurious detections caused by flight noise. Echo arrival times at the right and left microphones ($\:{\tau\:}_{r}$, $\:{\tau\:}_{l}$) were then determined from the peak positions of the respective envelopes, and the distance to the obstacle $\:{r}_{obs}$ was calculated as

1 $$\:\begin{array}{c}{r}_{obs}=\frac{c\left({\tau\:}_{r}+{\tau\:}_{l}\right)/2}{2}\end{array},$$

where *c* indicates sound velocity (= 340 m/s at ~ 15* ℃*). To estimate the horizontal obstacle direction while preserving the signal-to-noise ratio (SNR), the interaural time delay ( $\:\varDelta\:{\tau\:}_{rl}={\tau\:}_{r}-$ $\:{\tau\:}_{l}$, Fig. 1B) was calculated from independently estimated echo arrival times using the transmitted FM pulse as a reference signal, rather than by directly cross-correlating the two received echo signals. Specifically, the horizontal obstacle direction $\:{\theta\:}_{obs}$ was then estimated using the microphone separation *d* as follows:

2 $$\:\begin{array}{c}{\theta\:}_{obs}={\mathrm{sin}}^{-1}\left(\frac{c\varDelta\:{\tau\:}_{rl}}{d}\right)\end{array},$$

The azimuth estimation formula in Eq. (2) is computationally efficient and has been conventionally adopted in simple engineering sonar systems^10^. Detected obstacle distance $\:\left({r}_{obs}\right)$ and direction $\:\left({\theta\:}_{obs}\right)$ information were converted into steering direction command ($\:{\varphi\:}_{d}$) using our previously proposed 2D navigation model^37^ as follows:

3 $$\:\begin{array}{c}{\varphi\:}_{d}\left({t}_{s+1}\right)=arg\left({e}^{i{\varphi\:}_{d}\left({t}_{s}\right)}-2\sum\:_{n=0}^{N\left({t}_{s}\right)}\frac{\alpha\:}{{{r}_{obs}\:\left({t}_{s},\:n\right)}^{\rho\:}}\mathrm{sin}\left(\mathrm{arctan}\frac{\beta\:}{{r}_{obs}\left({t}_{s},\:n\right)}\right){e}^{i{\theta\:}_{obs}\left({t}_{s},\:n\right)}\right)\end{array},$$

where *N* and *n* indicate the number of localized obstacles and corresponding obstacle IDs, respectively. $\:{t}_{s}$ represents the discrete sensing time step. Briefly, the model computes an imaginary attractive force toward the present flight direction $\:{\varphi\:}_{d}\left({t}_{s}\right)$ and a imaginary repulsive force away from detected obstacles, and the resultant imaginary force vector determines the desired flight direction $\:{\varphi\:}_{d}\left({t}_{s+1}\right)$. Obstacle avoidance model in details was described in S1 Text. All computed values were stored and analyzed onboard Raspberry Pi. The generated heading command was transmitted to the flight controller through the onboard Raspberry Pi.

For autonomous navigation, an additional Raspberry Pi was installed as the flight controller, and linked to the sensing controller via LAN cable. Flight speed and inter-pulse interval were set to 0.4 m/s and 0.5 s, respectively. Cross-correlation processing and signal storage were implemented in C language, while LPM signal generation, serial communications, and flight control were programmed in Python.

### Echo detection test during hovering flight

**Fig. 2:**
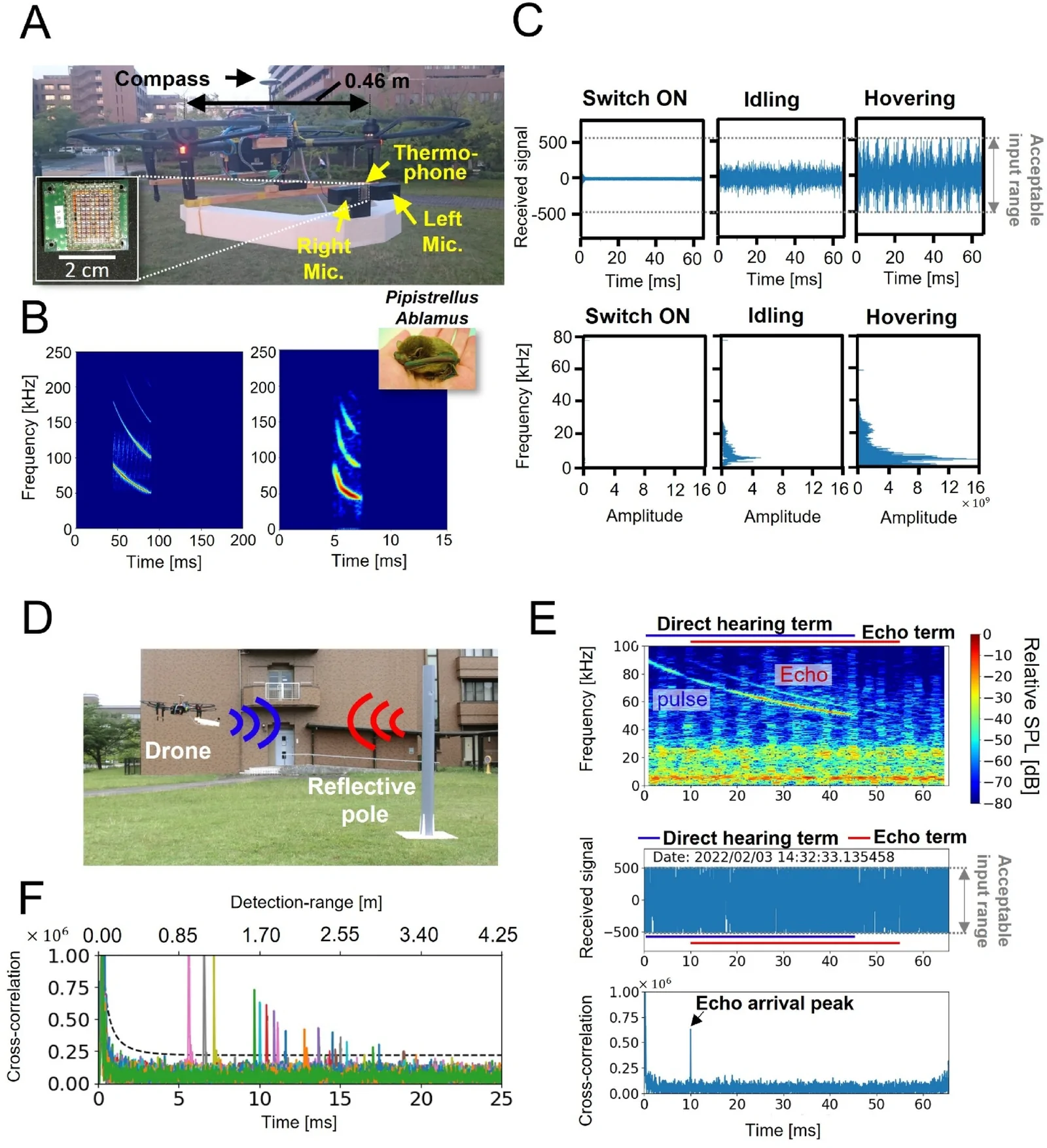
Demonstration of object detection using a drone-mounted ultrasonic sensing system during flight. (**A**) Customized drone equipped with a thermophone, two microphones, and an onboard processor. (**B**) Time-frequency structure of emission pulse mimicking the echolocation pulse of *Pipistrellus abramus*. (**C**) Representative noise signals and spectra recorded by the left microphone under three conditions: stationary without rotor motion (left), idling on the ground (center), and hovering (right), all without pulse emission. The amplitude fluctuations observed during hovering are likely associated with rotor-induced noise; however, the relationship between these fluctuations and rotor blade-passing frequencies remains to be investigated. (**D**) Experimental setup for target pole detection during hovering and approaching flight. (**E**) Example spectrogram, time-amplitude signal, and cross-correlation function obtained from the left receiver. (**F**) Echo peak strength after cross-correlation as a function of the target distance. Data were compiled from 23 sensing trials at different drone hovering positions. The black dashed line indicates the detection threshold.

Figure 2A shows proposed ultrasonic sensing system for drones based on the minimal echolocation design of bats: one transmitter and two receivers. Using a lightweight thermophone (6.6 g) as the ultrasonic transmitter, our system reproduced harmonic broadband FM pulses with a fundamental frequency sweep from 90 kHz to 50 kHz based on a linear periodic modulation (LPM) signal, similar frequency band to the echolocation pulse of the *Pipistrellus abramus* (Fig. 2B). Although the pulse duration (45 ms) is considerably longer than that of natural *Pipistrellus* echolocation calls, the comparison in Fig. 2B is intended to illustrate the similarity of the frequency-modulation structure. The longer duration was adopted to increase pulse-compression gain and improve echo detection robustness under strong drone flight noise. Driven by a 9 V dry cell battery, the root mean square level of the emission pulse was 1.4 Pa (97 dB SPL) at 0.05 m in front of the thermophone. In this study, three types of flight experiments were conducted with the onboard system to evaluate its performance during drone flight: (1) object detection, (2) object localization, and (3) obstacle avoidance.

At first, echo detection performance was evaluated during hovering flight. During hovering flight, drone-generated flight noise measured ~ 94 dB SPL at 0.7 m from the propeller. For reference, the emitted FM pulse had a sound pressure level of 97 dB SPL measured 0.05 m in front of the thermophone. Recordings from onboard MEMS microphones showed that the microphone signals were dominated by drone flight-induced noise (Fig. 2C, upper right), with spectral energy extending up to 30 kHz (Fig. 2C, lower right). Echo detection test was conducted with a polyvinyl pole (height = 2 m, diameter = 0.12 m) placed in front of the drone served as a target (Fig. 2D). Results confirmed that the sensing system could detect echoes reflected from the pole using cross-correlation process, despite the raw onboard microphone signals being dominated by drone flight-induced noise (Fig. 2E). The maximum detection distance was reached to 3.4 m (Fig. 2F). Object detection experiments were conducted over 80 independent pulse emissions, and objects were successfully detected in 67 of the 80 emissions.

### Experimental demonstration of 2D object mapping and obstacle avoidance during autonomous flight

**Fig. 3:**
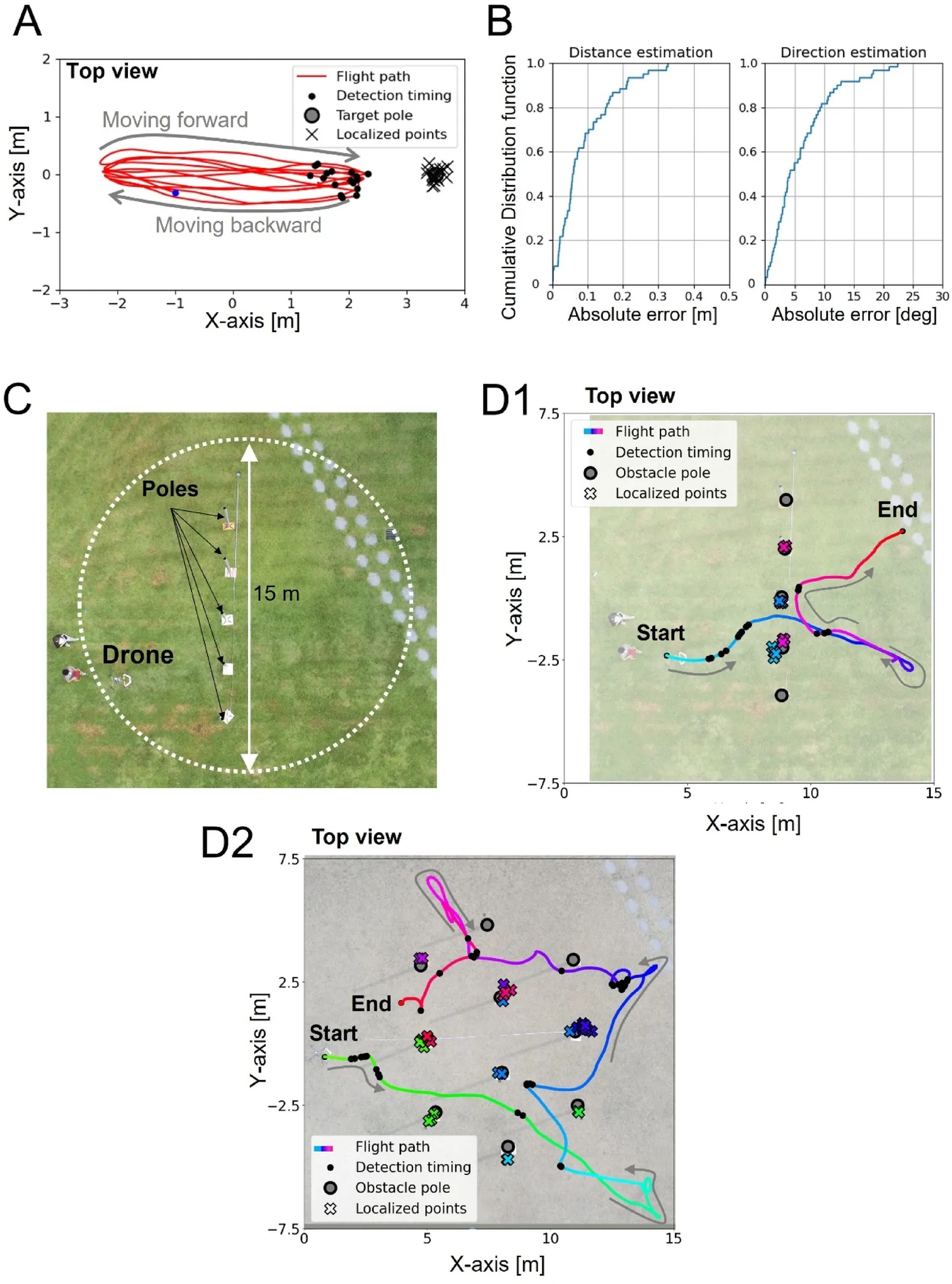
Demonstration of obstacle localization and avoidance by an autonomous drone equipped with the proposed ultrasonic sensing system. (**A**) Top views of localized target pole positions obtained during continuous flights in which the drone moved repeatedly between 1.5 and 5.5 m from the target. Grey circle denote that actual pole position, black crosses the detected positions, red line flight path, and black circles the echo acquisition points. (**B**) Cumulative distribution functions (CDF) of absolute localization errors based on 60 sensing points collected across 16 flights. (**C**) Top view of the test field (diameter = 15 m) acquired by aerial photography, showing the obstacle arrangement used for avoidance experiments. (**D**1-2) Flight paths and localized obstacle points during avoidance tests in two different obstacle configurations. Grey circles indicate obstacle positions, crosses the detected obstacle points, and rainbow-colored lines the flight paths (with synchronized timing).

In the next step, we evaluated object localization during continuous flight with a single pole. The target pole was manually placed 3.5 m in front of the drone along its initial heading direction using a measuring tape. The flight start position was defined as the origin, and this manually measured pole position was used as the ground-truth position. During flight, the drone position and heading were obtained from the DJI GPS/compass system, while the relative distance and azimuth angle to the pole were estimated by the ultrasonic sensing system. The estimated pole position in the initial coordinate frame was then calculated by integrating these measurements. Localization accuracy was evaluated by comparing the estimated and ground-truth pole positions. In this experiment, the drone autonomously moved back and forth between 1.5 and 5.5 m from the target pole while maintaining its heading toward the object (Fig. 3A). The flight speed was set at 0.4 m/s to ensure stable operation. As a result of 16 flight trials (16 forward movements and 16 backward movements), 346 pulses were emitted, from which 60 pole detections were obtained. Within a drone-to-pole distance of 2.5 m, 108 pulse emissions were transmitted, yielding a pole detection rate of 56% (60 detections from 108 pulse emissions). Furthermore, the pole was detected at least once during 15 of the 16 forward movements and 13 of the 16 backward movements. Localization errors were − 0.003 ± 0.12 m (mean ± SD) along the longitudinal axis (X) and 0.02 ± 0.21 m along the lateral axis (Y), corresponding to an average distance error of 0.09 ± 0.08 m and a directional error of 6.1 ± 5.2 degrees (Fig. 3B). The cumulative distributions in Fig. 3B further show that approximately 90% of localization estimates for the 12-cm-diameter pole used in this study were obtained within 0.2 m distance error and within 15° directional error. The mean flight speed during localization was 0.40 ± 0.15 m/s (mean ± SD).

Finally, as a proof-of-concept demonstration of the practical applicability of thermophone-based FM sonar on a UAV platform, we demonstrated autonomous obstacle avoidance by implementing a previous developed 2D avoidance model (see S1 text,^37^). The flight area was restricted to a 15-m-diameter circular field (Fig. 3C). Two different obstacle configurations were tested using multiple poles as shown in Fig. 3D. One trial was conducted for the first configuration (Fig. 3D-1), whereas four trials were conducted for the second configuration (Fig. 3D-2). In the first configuration, obstacle detections were confirmed in 13 of 144 emitted pulses during a 76 s flight. In the second configuration, obstacle detections were confirmed in 35 of 471 emitted pulses during a 227 s flight, 28 of 462 emitted pulses during a 184 s flight, 17 of 137 emitted pulses during a 56 s flight, and 49 of 233 emitted pulses during a 106 s flight. In one trial of the second configuration, the system failed to detect an obstacle pole located at a horizontal angle greater than 45° from the sensor’s forward direction, resulting in a collision with the pole due to crosswind in the 56 s flight trial. Figure 3D shows example avoidance flights under two different obstacle layouts (Fig. 3D; see Videos S1–S2). Localization accuracy was evaluated only for the two trials shown in Fig. 3D-1 and Fig. 3D-2, because the overhead images acquired in these trials were sufficiently stable for accurate trajectory reconstruction. Based on these two trials, the mean absolute localization error was 0.12 m (*n* = 48). In the example flights shown in Fig. 3D, avoidance turns were executed away from the nearest obstacle.

## Discussion

In this study, we developed a thermophone-based FM sonar system for UAV applications. Using a lightweight thermophone as the ultrasonic emitter, the proposed system generated broadband FM pulses comparable to those of bats (Fig. 2B), and demonstrated low-speed two-dimensional multi-obstacle avoidance flights in the horizontal plane using ultrasound alone during autonomous drone flight (Fig. 3D; Videos S1–S2). Future work will include direct comparisons with conventional ultrasonic sensing systems and controlled experiments to isolate the contribution of FM pulse design, thereby further evaluating the performance of thermophone-based FM sonar.

So far, most drone-mounted ultrasonic sensing systems employed constant-frequency signals^12,41^. Such signals are characterized by narrowband energy concentration, which is advantageous for the simple detection of distant reflectors, i.e., determining the presence or absence of objects at long range. On the other hand, broadband FM pulses provide high temporal resolution and facilitate matched-filter-based echo detection using correlation processing. By combining this signal design with a lightweight thermophone transmitter, the proposed onboard sonar system demonstrated two-dimensional localization and continuous avoidance of 12-cm-diameter poles using only two MEMS microphones. Considering the drone flight-induced noise level (94 dB SPL at 0.7 m), pulse emission level (97 dB SPL at 0.05 m), maximum detection range (3.4 m), and spreading loss, we roughly estimate that, under the experimental conditions used in this study, echoes may have been detectable at effective signal-to-noise ratios as low as − 30 dB. This value was not measured directly but inferred from these experimental parameters and should therefore be regarded as an approximate indication of the operating conditions rather than a quantitative evaluation of noise robustness. Throughout this study, the term *flight-induced noise* refers to the disturbance recorded by the onboard microphones during flight, which may include both acoustic noise generated by the rotors and electrical or mechanical noise originating from the drone platform. Their relative contributions were not separated in the present study. In these noisy conditions, the pole localization error remained within approximately 0.2 m at target distances of 1–3 m (Fig. 3A), provides a reference level for acoustic navigation in UAVs. Although the present study demonstrated reliable detection and localization of a 12-cm-diameter cylindrical pole, this target represents a relatively favorable reflector for ultrasonic sensing. Detection performance may vary for objects with different geometries, surface properties, and acoustic reflectivities, such as thin cables, vegetation, or irregular building structures. Future studies should therefore evaluate the proposed system not only for a wider variety of obstacle types but also under these more challenging and environmentally relevant conditions, such as fog or smoke, where the propagation characteristics of ultrasound may differ substantially from those under the clear conditions examined in the present study.

Importantly, the thermophone transmitter is extremely compact (25 × 30 × 10 mm, 6.6 g) and operates at a readily available voltage of only 9 V. Compared with conventional ultrasonic FM transmitters such as the SensComp (150–200 V, 4.7 g)^11,42,43^ and the VIFA XT25SC90-04 (~ 30 V_pp_, 70 g)^9,44^, the thermophone operates at a substantially lower voltage range directly compatible with standard onboard power supplies (e.g., Li-Po batteries), while remaining lightweight. As a result, it eliminates the need for dedicated high-voltage drive circuitry, thereby reducing the associated weight and volume overhead and lowering the practical barriers to implementing broadband FM sonar on small UAV platforms subject to strict payload and space constraints. The use of compact and readily deployable thermophones—operating with the wide bandwidth and lowest voltage input (approximately one-tenth of that required by conventional FM transmitters used on vehicles; Table 1)—makes thermophones a practical transmitter technology for airborne FM sonar systems. Rather than serving simply as an alternative ultrasonic emitter, thermophones provide a practical platform for implementing broadband FM sonar on lightweight UAVs and for investigating bat-inspired active sensing strategies during autonomous flight.

**Table 1:** Summary of related studies.

	[1] (2019)	[2] (2018)	[3] (2019)	[4] (2012)	[5] (2022)	[6] (2022)	[7] (2024)	This Work
Vehicle	AGV	AGV	AGV	Drone	Drone	Drone	Drone	Drone
TX/RX^1^	1/2	1/2	1/2	12/12	0/2	1/4	1/2	**1/2**
Tx mass	-	70 g	4.7 g	5 g	-	-	-	**6.6 g**
Tx input	-	~ 30 v	150 v	5 v	-	-	3.3 v	**9 v**
Ultrasonic FM pulse emission	×	✔ (20–100 kHz)	✔ (45 –20 kHz)	×	-	×	×	✔ (90 –50 kHz)
Direction of Echo Arrival	✔	✔	✔	×	✔	✔	×	✔
Full onboard	✔	✔	✔	✔	✔	×	✔	✔
Flight experiment	×	×	×	✔	✔	✔	✔	✔
2D avoidance	✔	✔	✔	× (1D)	×	× (1D)	✔	✔

TX/RX^1^: Number of Transmitter channel/ Number of Receiver channel.

[1] Bou Mansour et al.(2019)^8^, [2] Eliakim et al. (2018)^9^, [3] Laurijssen et al. (2019)^11^, [4] Gageik et al. (2012)^41^, [5] Zigelman et al. (2022)^44^, [6] Dümbgen et al. (2022)^45^, [7] Müller et al. (2024)^12^.

Our system adopts a simple and scalable signal processing architecture that can be implemented on embedded hardware such as a Raspberry Pi 4 (1.5 GHz quad-core CPU), without relying on deep learning or other computationally intensive methods. This design choice is amenable to the extension of previously proposed acoustic sensing techniques. For example, bio-inspired approaches reported in the literature, including SLAM adapted for biomimetic sonar^46^ and artificial acoustic landmarks^43^, may be incorporated. Other applications of ultrasonic sensing reported in the literature—such as vegetation mapping^9^, leaf density estimation^42^, and gap detection in dense vegetation^38^—could also be considered for extension from ground-based platforms to UAVs using the proposed sonar system. Although the present study focused on obstacle avoidance, the proposed framework provides a basis for extending onboard ultrasonic sensing to other bat-inspired behaviors, including target pursuit.

From a biological perspective, the design of echolocation pulses and associated signal processing in bats remains an active research topic. Behavioral studies show that bats flexibly adjust pulse duration, bandwidth, and inter pulse interval depending on task demands^35,47,48^. For example, bats lengthen terminal pulse segments when searching for distant prey, shorten intervals during close approaches, or broaden frequency bands to improve resolution^18,35^. These observations suggest that adaptive modulation of sonar signals contributes to behavioral flexibility in bats. Because thermophones provide a wide, nearly flat frequency response (within a 10 dB range up to 120 kHz), they offer a practical hardware platform for implementing adaptive pulse control algorithms inspired by bat echolocation. Future work will therefore investigate real-time adaptation of pulse parameters, including bandwidth, duration, and pulse repetition interval, to evaluate bat-inspired active sensing strategies during autonomous flight.

## Materials and methods

### A thermophone as a wideband ultrasonic transmitter

**Fig. 4:**
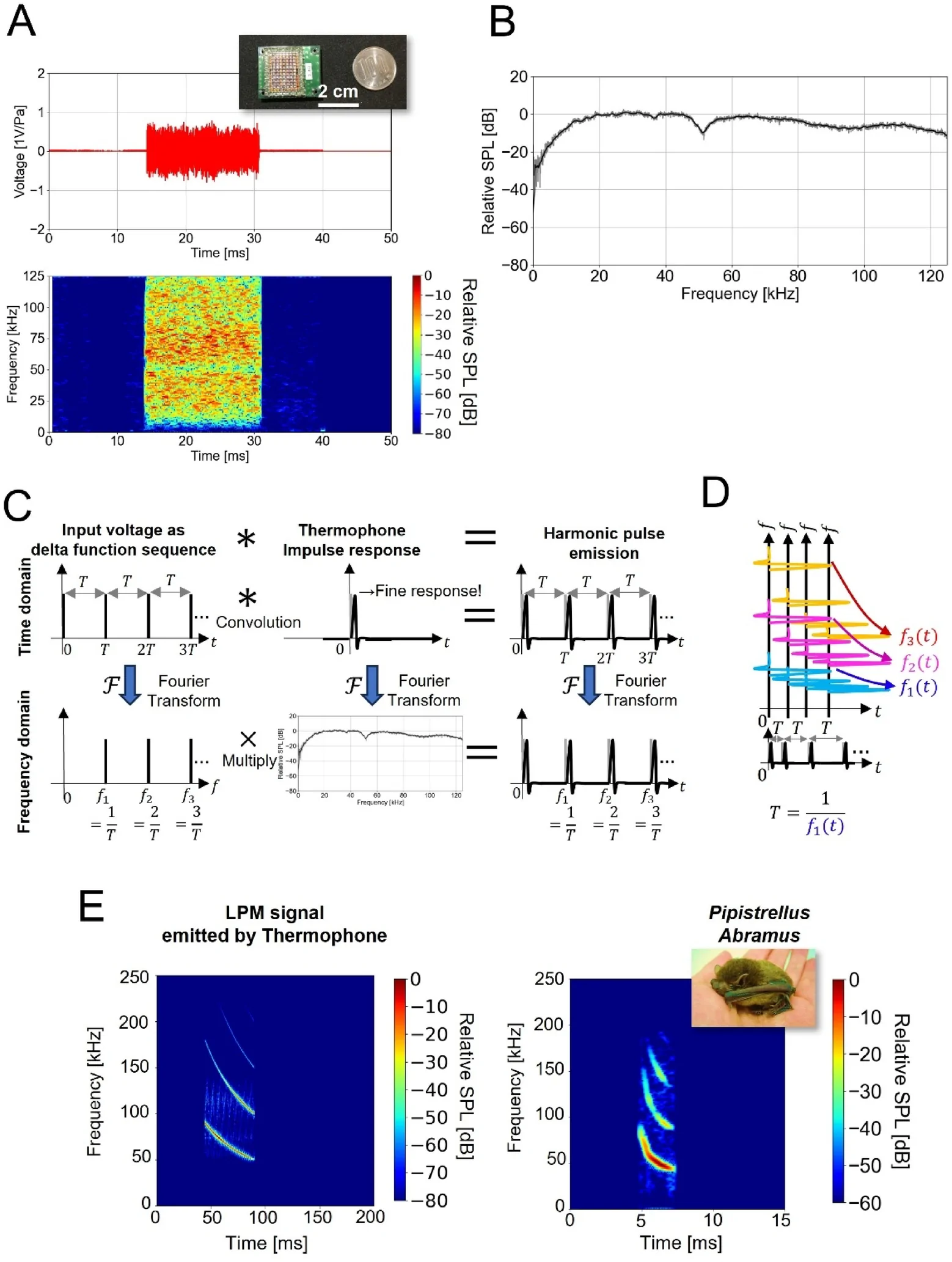
Reproducing method for harmonic echolocation call of FM bats utilizing the thermophone with flat frequency response. (**A**) Time-amplitude waveform and spectrogram of the emission pulse when 12-bit M-sequence signal was input to the thermophone. The thermophone was powered by a 9v dry cell and recorded with the microphone positioned 0.05 m in front of the thermophone at 250 kHz sampling rate. (**B**) Frequency response of the thermophone, evaluated with 12-bit M-sequence signal. The grey line shows the additive average of eight emissions, and the black line indicates the response after applying a 20-points moving average. (**C**) Schematic diagram of harmonic pulse emission with an impulse sequence. The upper and lower panels show signal dynamics in the time and the frequency domains, respectively. (**D**) Schematic time-amplitude waveform and spectrogram of a harmonic down-sweeping FM pulse constructed with an impulse sequence. The inter-impulse interval * T* matches the instantaneous fundamental frequency period of the FM pulse $\:{f}_{1}\left(t\right)$. (E) Spectrogram of harmonic FM pulses emitted by the thermophone and by *Pipistrellus Abrams*. The down-sweep FM pulses from the thermophone was encoded by a Linear Periodic Modulation (LPM) signal with fundamental frequencies set from 90 kHz to 50 kHz. Recordings were obtained with the microphone positioned 0.05 m in front of the thermophone at a 1 MHz sampling rate.

Thermophones are the thermoacoustic transducer that emit sound via air vibration caused by periodic heating and cooling of a metal plate synchronized with the input power^39,40^. Because no mechanical resonance is involved, thermophones can generate broadband signals. Our proposed thermophone (Fig. 4A) produced broadband pulses (12bit M-series signal) recorded with a 1/4-inch condenser microphone (Brüel and Kjaer, Model 4939-A) at 0.05 m in front of the emitter. With 9 V input, the root mean square (RMS) sound pressure was 0.28 Pa, corresponding to approximately 83 dB SPL. Although the measurement system limited evaluation to 120 kHz, the frequency response was flat to within 10 dB between 10 and 120 kHz (Fig. 4B), indicating suitability for reproducing FM bat calls. FM bats emit harmonic down-sweep FM pulses, with fundamental frequency dropping from approximately 100 kHz to several tens of kHz^26,27^. To reproduce harmonic non-linear FM signals, we used impulse sequences in which the inter-impulse interval was matched to the instantaneous period of the FM pulse in a fundamental frequency component (Fig. 4C, D). The flat frequency response of the thermophone allowed accurate reproduction of harmonic down-sweep pulses (Fig. 4D).

Pulses of vespertilionid bats have been well known to exhibit non-linear frequency modulation, often well approximated by exponential FM rather than strict linear modulation, characterized by a steep frequency sweep at high frequencies followed by a shallower sweep at lower frequencies^49^. Motivated by this structural property, we encoded linear period modulated (LPM) signal into the transmitted impulse sequence as an analytically tractable representative of such non-linear FM waveforms, for which Doppler tolerance of cross-correlation peaks is theoretically guaranteed in mobile sensing scenarios^50^. The LPM signal $\:S\left(t\right)\:$was defined as

4 $$\:\begin{array}{c}S\left(t\right)=\mathrm{sin}\left(2\pi\:{f}_{i}\mathrm{log}\left(\frac{{f}_{i}-{f}_{t}}{{f}_{t}{t}_{d}}t+1\right)\right),\:t\in\:\left[0,\:{t}_{d}\right] \end{array}$$

where $\:{f}_{i}$ and $\:{f}_{t}$ indicate the initial and terminal frequencies of the LPM signal (90 kHz and 50 kHz, respectively), and $\:{t}_{d}$ denotes the pulse duration (45 ms). Impulse sequences were generated by inducing impulses at positive peaks of $\:S\left(t\right)$, corresponding to a 90-degree phase. As a result, harmonic down-sweep FM pulse was represented as shown in Fig. 4E. The RMS level of the emission pulse was 1.4 Pa (~ 97 dB SPL at 0.05 m in front of the thermophone).

**Fig. 5:**
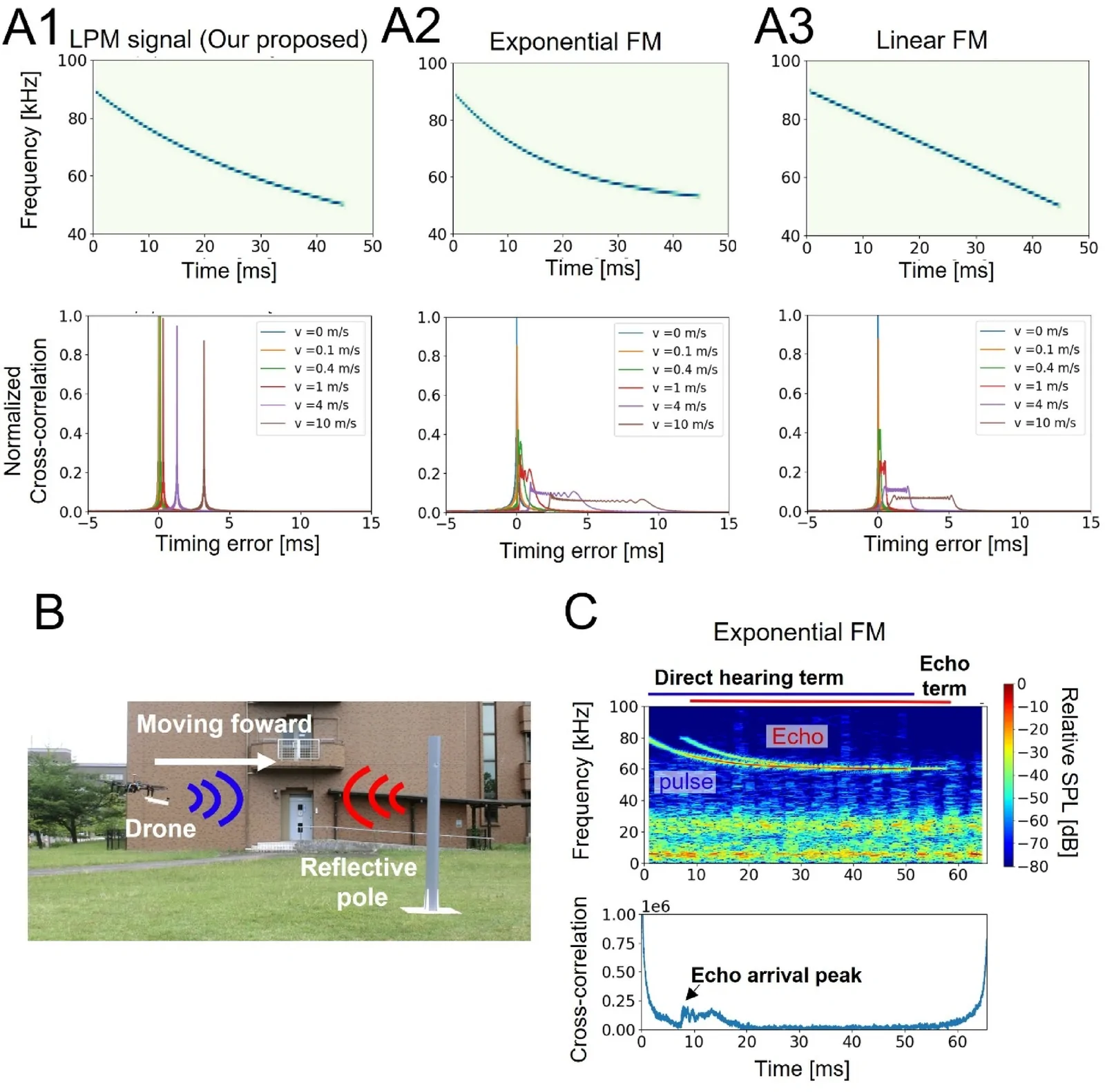
LPM signal superiority in the presence of negative Doppler shift effects, demonstrated by experimental results and mathematical simulations. (**A**) Mathematical simulations showing the deformation of cross-correlation signals due to Doppler shifts, as a function of flight speed. These simulations were conducted using an LPM signal (A1 panels), an exponential FM (A2 panels), and a linear-FM signal (A3 panels), respectively. In the simulations applying exponential FM and linear-FM signals, cross-correlation peaks decreased with increasing flight speed, while the LPM signal exhibited strong cross-correlation peaks. (**B**) A demonstration of drone sensing during approaching flight toward to a pole. (**C**) A typical spectrogram and cross-correlation signal for pulse and echo during approaching flight experimented with an exponential FM model. The cross-correlation of the echo signal was quite weak and distorted due to Doppler shift effects. In addition, the deformation of cross-correlation signal was appeared to be well represented by the simulated signal (see A-2), such that Doppler shift effects had a negative influence on cross-correlation peaks in the echo. This analysis indicates that LPM signals are robust against Doppler shift effects and are thus well suited for mobile sensing systems.

The Doppler tolerance of cross-correlation peak strength during approach to an object was examined theoretically by numerical simulations using LPM, exponential FM^49^, and linear FM signals (Fig. 5A1–A3). These simulations confirmed that the LPM signal exhibits robustness to Doppler shift effects under motion. In actual experiments using an exponential FM signal, cross-correlation peak reduction was also observed due to the waveform distortion consistent with the simulation (Fig. 5B, C). Based on this agreement between simulation and experiment, we concluded that the LPM signal is effective for the experiments considered in this study.

## Experimental setup

Flight experiments were conducted on the Higashi-Hiroshima campus of Hiroshima University. Multiple vinyl chloride poles (height: 2.0 m, diameter: 0.12 m) were placed as obstacles and target objects. Hovering tests were conducted under manual control using a remote controller, while all other experiments employed autonomous flights programmed via the DJI onboard SDK and Robot Operating System (ROS). Video recordings of avoidance flights were acquired using an additional drone (Parrot, Anafi) flying at ~ 30 m altitude above the test field. In compliance with Japanese regulations, all drones were registered with the Drone/UAS Information Platform System, operated by the Ministry of Land, Infrastructure, Transport and Tourism (Experimental drone ID: JU322A4B9C54, Movie drone ID: JU322AFFEDC0). Actual 2D flight paths and pole positions were extracted from overhead video using the open-source tracking software UMA Tracker^51^. To correct for image jitter caused by flight motion and wind, video frames were translated and rotated to align pole position consistently. Flight path and pole positions were then synchronized with onboard compass and ultrasonic localization data using timestamps.

## Supplementary Information

Below is the link to the electronic supplementary material.

Supplementary Material 1

Supplementary Material 2

Supplementary Material 3

Supplementary Material 4

## Data Availability

All raw data are available at https://doi.org/10.5281/zenodo.8082146.

## References

[1] Lu, Y., Xue, Z., Xia, G. S. & Zhang, L. A survey on vision-based UAV navigation. *Geo-spatial Inform. Sci.* **21**, 21–32 (2018).

[2] Yang, H., Lee, Y., Jeon, S. Y. & Lee, D. Multi-rotor drone tutorial: systems, mechanics, control and state estimation. *Intel. Serv. Robot.* **10**, 79–93 (2017).

[3] Zhou, X. et al. Swarm of micro flying robots in the wild. *Sci. Rob.* **7**, eabm5954 (2022). 10.1126/scirobotics.abm5954 35507682

[4] Kendoul, F., Fantoni, I. & Nonami, K. Optic flow-based vision system for autonomous 3D localization and control of small aerial vehicles. *Robot. Auton. Syst.* **57**, 591–602 (2009).

[5] Keshavan, J., Gremillion, G., Alvarez-Escobar, H. & Humbert, J. S. Autonomous vision-based navigation of a quadrotor in corridor-like environments. *Int. J. Micro Air Veh.* **7**, 111–123 (2015).

[6] Sanket, N. J. et al. Active vision based minimalist structure-less gap detection for quadrotor flight. *IEEE Rob. Autom. Lett.* **3**, 2799–2806 (2018).

[7] de Croon, G. C., De Wagter, C. & Seidl, T. Enhancing optical-flow-based control by learning visual appearance cues for flying robots. *Nat. Mach. Intell.* **3**, 33–41 (2021).

[8] Bou Mansour, C., Koreman, E., Steckel, J., Peremans, H. & Vanderelst, D. Avoidance of non-localizable obstacles in echolocating bats: A robotic model. *PLoS Comput. Biol.* **15**, e1007550 (2019). 31856162 10.1371/journal.pcbi.1007550 PMC6941896

[9] Eliakim, I., Cohen, Z., Kosa, G. & Yovel, Y. A fully autonomous terrestrial bat-like acoustic robot. *PLoS Comput. Biol.* **14**, e1006406 (2018). 30188901 10.1371/journal.pcbi.1006406 PMC6126821

[10] Velmurugan, M., Brush, P., Balfour, C., Przybyla, R. J. & Sanket, N. J. Milliwatt ultrasound for navigation in visually degraded environments on palm-sized aerial robots. *Sci. Rob.* **11**, eadz9609 (2026). 10.1126/scirobotics.adz9609 41880528

[11] Laurijssen, D., Kerstens, R., Schouten, G., Daems, W. & Steckel, J. In *2019 International Conference on Robotics and Automation (ICRA).*. 9617–9623 (IEEE).

[12] Müller, H., Kartsch, V., Magno, M. & Benini, L. In *2024 IEEE Sensors Applications Symposium (SAS).* 1–6 (IEEE).

[13] Griffin, D. R. Listening in the dark: the acoustic orientation of bats and men. (1958).

[14] Neuweiler, G. & Foraging Echolocation and Audition in Bats. *Naturwissenschaften* **71**, 446–455 (1984).

[15] Surlykke, A., Ghose, K. & Moss, C. F. Acoustic scanning of natural scenes by echolocation in the big brown bat, *Eptesicus fuscus*. *J. Exp. Biol.* **212**, 1011–1020 (2009). 19282498 10.1242/jeb.024620 PMC2726860

[16] Teshima, Y., Yamada, Y., Tsuchiya, T., Heim, O. & Hiryu, S. Analysis of echolocation behavior of bats in echo space using acoustic simulation. *BMC Biol.* **20**, 1–12 (2022). 35282831 10.1186/s12915-022-01253-y PMC8919609

[17] Yamada, Y. et al. Modulation of acoustic navigation behaviour by spatial learning in the echolocating bat Rhinolophus ferrumequinum nippon. *Sci. Rep.* **10**, 1–15 (2020). 32612132 10.1038/s41598-020-67470-z PMC7329871

[18] Jakobsen, L. & Surlykke, A. Vespertilionid bats control the width of their biosonar sound beam dynamically during prey pursuit. *Proc. Natl. Acad. Sci.* **107**, 13930–13935 (2010). 20643943 10.1073/pnas.1006630107 PMC2922241

[19] Ghose, K., Horiuchi, T. K., Krishnaprasad, P. S. & Moss, C. F. Echolocating bats use a nearly time-optimal strategy to intercept prey. *PLoS Biol.* **4**, e108. 10.1371/journal.pbio.0040108 (2006). 16605303 PMC1436025

[20] Fujioka, E. et al. Rapid shifts of sonar attention by *Pipistrellus abramus* during natural hunting for multiple prey. *J. Acoust. Soc. Am.* **136**, 3389–3400 (2014). 25480083 10.1121/1.4898428

[21] Fujioka, E., Aihara, I., Sumiya, M., Aihara, K. & Hiryu, S. Echolocating bats use future-target information for optimal foraging. In: *Proceedings of the National Academy of Sciences*, 201515091 (2016). 10.1073/pnas.1515091113 PMC4855569 27071082

[22] Tian, B. & Schnitzler, H. U. Echolocation signals of the greater horseshoe bat (Rhinolophus ferrumequinum) in transfer flight and during landing. *J. Acoust. Soc. Am.* **101**, 2347–2364 (1997). 9104033 10.1121/1.418272

[23] Habersetzer, J. Adaptive echolocation sounds in the bat Rhinopoma hardwickei: a field study. *J. Comp. Physiol.* **144**, 559–566 (1981).

[24] Hase, K. et al. Bats enhance their call identities to solve the cocktail party problem. *Commun. Biol.*. **1**, 39 (2018). 10.1038/s42003-018-0045-3 PMC6123623 30271924

[25] Hase, K., Kadoya, Y., Takeuchi, Y., Kobayasi, K. I. & Hiryu, S. Echo reception in group flight by Japanese horseshoe bats, Rhinolophus ferrumequinum nippon. *Royal Soc. Open. Sci.* **9**, 211597 (2022). 10.1098/rsos.211597 PMC8825988 35154795

[26] Fenton, M. B., Faure, P. A. & Ratcliffe, J. M. Evolution of high duty cycle echolocation in bats. *J. Exp. Biol.* **215**, 2935–2944 (2012). 22875762 10.1242/jeb.073171

[27] Fenton, M. B. & Bell, G. P. Recognition of species of insectivorous bats by their echolocation calls. *J. Mammal.* **62**, 233–243 (1981).

[28] Surlykke, A. & Kalko, E. K. Echolocating bats cry out loud to detect their prey. *PLoS one*. **3**, e2036 (2008). 18446226 10.1371/journal.pone.0002036 PMC2323577

[29] Schnitzler, H. U. & Grinnell, A. Directional sensitivity of echolocation in the horseshoe bat, Rhinolophus ferrumequinum. *J. Comp. Physiol.* **116**, 51–61 (1977).

[30] Yamada, Y., Hiryu, S. & Watanabe, Y. Species-specific control of acoustic gaze by echolocating bats, *Rhinolophus ferrumequinum nippon* and *Pipistrellus abramus*, during flight. *J. Comp. Physiol. A.* **202**, 791–801 (2016). 10.1007/s00359-016-1121-0 PMC5061877 27566319

[31] Hartley, D. J. & Suthers, R. A. Sonar pulse radiation and filtering in the mustached bat, Pteronotusparnelliirubiginosus. *J. Acoust. Soc. Am.* **87**, 2756–2772 (1990).

[32] Hartley, D. J. & Suthers, R. A. The sound emission pattern of the echolocating bat, Eptesicusfuscus. *J. Acoust. Soc. Am.* **85**, 1348–1351 (1989). 10.1121/1.395684 3429728

[33] Jakobsen, L., Ratcliffe, J. M. & Surlykke, A. Convergent acoustic field of view in echolocating bats. *Nature* **493**, 93–96. 10.1038/nature11664 (2013). 23172147

[34] Schnitzler, H. U. & Denzinger, A. Auditory fovea and Doppler shift compensation: adaptations for flutter detection in echolocating bats using CF-FM signals. *J. Comp. Physiol. A.* **197**, 541–559 (2011). 10.1007/s00359-010-0569-6 20857119

[35] Hiryu, S., Hagino, T., Fujioka, E., Riquimaroux, H. & Watanabe, Y. Adaptive echolocation sounds of insectivorous bats, *Pipistrellus abramus*, during foraging flights in the field. *J. Acoust. Soc. Am.* **124**, EL51–EL56 (2008). 18681502 10.1121/1.2947629

[36] Matsuta, N. et al. Adaptive beam-width control of echolocation sounds by CF-FM bats, *Rhinolophus ferrumequinum nippon*, during prey-capture flight. *J. Exp. Biol.* **216**, 1210–1218. 10.1242/jeb.081398 (2013). 23487269

[37] Yamada, Y. et al. Ultrasound navigation based on minimally designed vehicle inspired by the bio-sonar strategy of bats. *Adv. Robot.*, 1–14 (2019).

[38] Wang, R., Liu, Y. & Müller, R. Detection of passageways in natural foliage using biomimetic sonar. *Bioinspir. Biomim.* **17**, 056009 (2022). 10.1088/1748-3190/ac7aff 35728778

[39] Shinoda, H., Nakajima, T. & Ueno, K. Koshida, N. Thermally induced ultrasonic emission from porous silicon. *Nature* **400**, 853–855 (1999).

[40] Nishioka, T. et al. Ultrasound radiation from a three-layer thermoacoustic transformation device. *Ultrasonics* **57**, 84–89 (2015). 25465964 10.1016/j.ultras.2014.10.019

[41] Gageik, N., Müller, T. & Montenegro, S. Obstacle detection and collision avoidance using ultrasonic distance sensors for an autonomous quadrocopter. *University Wurzburg Aerosp. Inform. Technologhy (germany) Wurzburg*, 3–23 (2012).

[42] Ming, C., Gupta, A. K., Lu, R. & Zhu, H. Müller, R. A computational model for biosonar echoes from foliage. *PLoS One*. **12**, e0182824 (2017). 28817631 10.1371/journal.pone.0182824 PMC5560571

[43] de Backer, M. et al. *2023 IEEE Sensors* 1–4 (IEEE).

[44] Zigelman, R., Eitan, O., Mazar, O., Weiss, A. & Yovel, Y. A bio-mimetic miniature drone for real-time audio based short-range tracking. *PLoS Comput. Biol.* **18**, e1009936 (2022). 35259156 10.1371/journal.pcbi.1009936 PMC8932603

[45] Dümbgen, F., Hoffet, A., Kolundžija, M., Scholefield, A. & Vetterli, M. Blind as a bat: Audible echolocation on small robots. *IEEE Rob. Autom. Letters* (2022).

[46] Steckel, J., Peremans, H. & BatSLAM Simultaneous localization and mapping using biomimetic sonar. *PLoS One*. **8**, e54076 (2013). 23365647 10.1371/journal.pone.0054076 PMC3554696

[47] Moss, C. F. & Surlykke, A. Probing the natural scene by echolocation in bats. *Front. Behav. Neurosci.* **4**, 33 (2010). 20740076 10.3389/fnbeh.2010.00033 PMC2927269

[48] Schnitzler, H. U., Moss, C. F. & Denzinger, A. From spatial orientation to food acquisition in echolocating bats. *Trends Ecol. Evol.* **18**, 386–394 (2003).

[49] Boonman, A. & Schnitzler, H. U. Frequency modulation patterns in the echolocation signals of two vespertilionid bats. *J. Comp. Physiol. Neuroethol Sens. Neural Behav. Physiol.* **191**, 13–21. 10.1007/s00359-004-0566-8 (2005). 15568143

[50] Yang, J. & Sarkar, T. Doppler-invariant property of hyperbolic frequency modulated waveforms. *Microw. Opt. Technol. Lett.* **48**, 1174–1179 (2006).

[51] Yamanaka, O. & Takeuchi, R. UMATracker: an intuitive image-based tracking platform. *J. Exp. Biol.* **221**, jeb182469 (2018). 29954834 10.1242/jeb.182469

