# Supplementary material for: A thermophone-based FM sonar for airborne ultrasonic sensing under drone flight noise
Source: Sci Rep. 2026 Sep 10;16:26651. doi: 10.1038/s41598-026-65909-3 (PMC13562668; doi:10.1038/s41598-026-65909-3)
Supplement: Supplementary file 1 [file 41598_2026_65909_MOESM1_ESM.pdf]

S1 Text. The obstacle avoidance navigation system implemented on the Drone.

In this study, an obstacle avoidance system was constructed by referencing previously proposed 2D obstacle avoidance models indicated by Yamada et al. in 2019, to produce a multi-obstacle-avoidance (MOA) system. This customized MOA model is capable of calculating the avoidance direction φ_d for the next $(s+1)$ agents using distance (r) and direction (θ) information for obstacles localized in the sth sensing step as follows:

$$\varphi_d(t_{s+1}) = \arg\left(e^{i\varphi_d(t_s)} - 2\sum_{n=0}^{N(t_s)} \frac{\alpha}{r(t_s,n)^{\rho}} \sin\left(\arctan\frac{\beta}{r(t_s,n)}\right) e^{i\theta(t_s,n)} \right),$$

where N and n indicate the number of localized obstacles and corresponding obstacle IDs, respectively. This system is constructed from a potential model, in which the avoidance direction is calculated as the sum of attractive and repulsive force vectors. The attractive vector is generated from the current agent's head direction as $e^{i\varphi_d(t_s)}$, while the repulsive vector is generated opposite the direction of each localized obstacle as $-e^{i\theta(t_s,n)}$. Note the length of repulsive force vectors corresponds to inverse ρ-square law of the distance of each obstacle, while $\alpha, \beta,$ and ρ are parameters used to determine repulsive force strength criteria. To turn more than 90 deg when the drone detects the obstacle within the 1.4 m, these values were set to 1800, 0.8, and 0.001, respectively. This approach and

parameter meanings were described in detail by Yamada et al. in 2019. The flight speed v was also controlled using the proposed model as follows:

$$v = (v_{max} - v_{min})e^{-{\varphi_d}^2} + v_{min},$$

where v_{max} and v_{min} indicate the desired maximum (typical) and minimum flight speed parameters. This constraint allows flight speed to correspond with the avoidance turn angle φ_d, such that flight speed decreases when a quick avoidance turn is required. Note the maximum (typical) and minimum flight speeds were set to 0.4 m/s and 0.05 m/s, respectively. However, the drone moved continuously due to inertial forces during actual flights. The proposed obstacle avoidance navigation system was constructed from the corresponding direction and flight speed control requirements.
