# Supplementary material for: A thermophone-based FM sonar for airborne ultrasonic sensing under drone flight noise
Source: Sci Rep. 2026 Sep 10;16:26651. doi: 10.1038/s41598-026-65909-3 (PMC13562668; doi:10.1038/s41598-026-65909-3)
Supplement: Supplementary file 2 [file 41598_2026_65909_MOESM4_ESM.docx]

Supporting Information

**S1-2 Video.** Demonstration movies for the obstacle avoidance navigation by an our proposed autonomous flying Drone.
